# Molecular diversity of Diplura in southern High Appalachian leaf litter

**DOI:** 10.3897/BDJ.12.e125162

**Published:** 2024-05-27

**Authors:** Ernesto Recuero, Michael S. Caterino

**Affiliations:** 1 Clemson University, Clemson, United States of America Clemson University Clemson United States of America

**Keywords:** Diplura, Hexapoda, soil biodiversity, megabarcoding, Appalachia, species delimitation, genetic distance, COI

## Abstract

The fauna of Diplura, the two-pronged bristletails (Hexapoda), of the southern Appalachians has received little focused systematic attention. Existing literature suggests the fauna to comprise around a dozen species. Based on a broader DNA barcode-based survey of high elevation litter arthropods in the region, we suggest the fauna to be much richer, with automated species delimitation methods hypothesising as many as 35 species, most highly restricted to single or closely proximate localities. Such a result should not be very surprising for such small, flightless arthropods, although it remains to be seen if other markers or morphology support such high diversity. The region still remains sparsely sampled for these more cryptic elements of the arthropod fauna and much larger numbers of species undoubtedly remain to be discovered.

## Introduction

The Diplura, commonly known as two-pronged bristletails, is a small group of Hexapoda with no clear phylogenetic relationships, being frequently classified as an order within the class Entognatha or as its own class more closely related to Insecta than to Protura and Collembola (Tihelka et al. 2021). There are around 1000 species in 141 genera, distributed worldwide, except Antarctica and many oceanic islands (Sendra et al. 2021). Currently 10 families are recognised, although most species diversity belongs to Campodeidae Meinert, 1865 (49% of all species) and Japygidae Haliday, 1864 (34%) (Sendra et al. 2021). They frequently live in hypogean habitats, including different soil strata and caves, but they can also be found in leaf litter, mosses, under rocks and tree bark, epiphytic vegetation and living in dead wood, always associated with high humidity microhabitats (Palacios-Vargas and García-Gómez 2014, Sendra et al. 2021), with species found from intertidal areas at sea level (Bu et al. 2012) to almost 5000 m in the Himalayas (Condé and Nguyen Duy-Jacquemin 1968).

The diversity of Diplura in North America is still considered understudied (Graening et al. 2014, Palacios-Vargas and García-Gómez 2014, Sikes 2019). The work of taxonomists such as F. Silvestri, B. Condé and of L. M. Smith during the 20^th^ century established the foundations of our knowledge of North American Diplura diversity (e.g. Silvestri 1911, Silvestri 1933, Silvestri 1947, Silvestri 1948, Condé and Thomas 1957, Condé and Bareth 1958, Smith 1959, Smith and Bolton 1964, Condé 1973). Allen (2002) provided a detailed catalogue for the Diplura of this continent, including identification keys to genera and information on the known distribution of species, to which only a few new species have been added during the last two decades (e.g. Allen (2003), Allen (2005), Allen (2006), Castaño Meneses and García Gómez (2007), García-Gómez (2009), García-Gómez (2016), Sendra et al. (2016), Montejo-Cruz et al. (2021)). To date, almost 200 species are known north of the Isthmus of Tehuantepec, including some potentially introduced species like *Parajapyxisabellae* (Grassi, 1886) or *Campodeapempturochaeta* Silvestri, 1912 (Graening et al. 2014, Sendra and Moreno 2004). More specifically, in the south-eastern USA (VA, NC, SC, TN, GA, FL, AL, MS, LA), there are 37 species representing 11 different genera and three families, Campodeidae (19 spp), Japygidae (13 spp) and Parajapygidae Womersley, 1939 (5 spp) (Allen 2002, Allen 2003, Allen 2005, Allen 2006). Of these, at least 12 species have been reported from the bulk of the southern Appalachian Mountains, from south-western Virginia to northern Georgia, six in the Campodeidae and six in the Japygidae, although several more are known from nearby areas and the diversity in these mountains is likely higher (Smith and Bolton 1964, Reddell 1983, Allen 1994, Allen 2002, Allen 2003, Allen 2005, Allen 2006). Here, we analyse the results for Diplura of a barcoding project aimed at characterising the arthropod fauna living in leaf litter habitats from high-elevation forests in the southern Appalachians (Caterino and Recuero 2024), though sampling includes also several mid-elevation localities. Despite having only family-level identifications, the use of molecular barcodes and species delimitation methods allow us to provide a first overview of the diversity of this small and fascinating group of hexapods in one of the most diverse regions in temperate North America.

## Material and methods

The methodology has been described in detail in previous papers (Caterino and Recuero 2023, Caterino and Recuero 2024, Recuero and Caterino 2024a). Sampling was performed twice per locality, in spring and autumn, by sifting three leaf litter subsamples at each locality, which were subsequently processed using Berlese funnels. Localities included all high-elevation fir-spruce forests in the southern Appalachian Mountains and several lower-elevation sites including deciduous and *Rhododendron* L. litters. Specimens were sorted by morphotypes and one specimen per morphotype from each locality and sampling event was selected for molecular analyses. Each selected specimen was photographed before DNA extraction (photos available at https://www.flickr.com/photos/183480085@N02/albums/72157710333788597/).

For DNA extraction, we digested the whole specimens with proteinase K and the remaining exoskeleton was recovered when possible, preserved in 95% ethanol with a drop of propylene glycol and deposited at the Clemson University Arthropod Collection (http://www.cuacinsects.org) for future morphological study. DNA was extracted with the Mag-Bind HDQ Blood and Tissue Kit (Omega BioTek). A minibarcode fragment of the Cytochrome c oxidase I (COI) mitochondrial gene was amplified via polymerase chain reaction (PCR) using the primer pair BF2-BR2 (Elbrecht and Leese 2017), with an annealing temperature of 50ºC. Primers were indexed with individual 9 bp tags to allow multiplexed sequencing (Meier et al. 2016). PCR products were mixed, purified and sequenced together using Illumina or Nanopore platforms.

Aligned sequences were analysed with PAUP v.4.0a (Swofford 2003) to produce a Neighbour-joining (NJ) tree, based on Kimura 2-parameter (K2P) distances (Kimura 1980), illustrating the genetic distances amongst the barcoded samples. This NJ tree was rooted with an acerentomid proturan (GenBank accession number OR171392, Caterino and Recuero (2024)). We used Assemble Species by Automatic Partitioning (ASAP, available at https://bioinfo.mnhn.fr/abi/public/asap/; Puillandre et al. (2021)) to generate preliminary hypotheses of species richness, based again on K2P distances. We estimated mean inter- and intraspecific uncorrected p-distances using MEGA v.11 (Tamura et al. 2021). Our preliminary family-level identification was confirmed by aligning our barcodes with all Diplura COI sequences available to date from GenBank and Barcode of Life Data System (BOLD) and performing Maximum Likelihood (ML) phylogenetic analyses to estimate their phylogenetic relationships. For this, we used W-IQ-Tree (Trifinopoulos et al. 2016) (available at http://iqtree.cibiv.univie.ac.at), with a GTR+F+I+G4 substitution model (automatically estimated at the beginning of the analysis under the Bayesian Information Criterion) and 1000 ultrafast bootstrap replicates to measure branch support of the obtained phylogenetic reconstruction.

## Data resources

This paper provides as data resources a sequence alignment in nexus format including all new Diplura barcodes (Suppl. material 1), as well as a file in Excel format including all metadata associated with vouchers and their unique identifiers and GenBank codes (Suppl. material 2).

## Results

**Figure 1.**
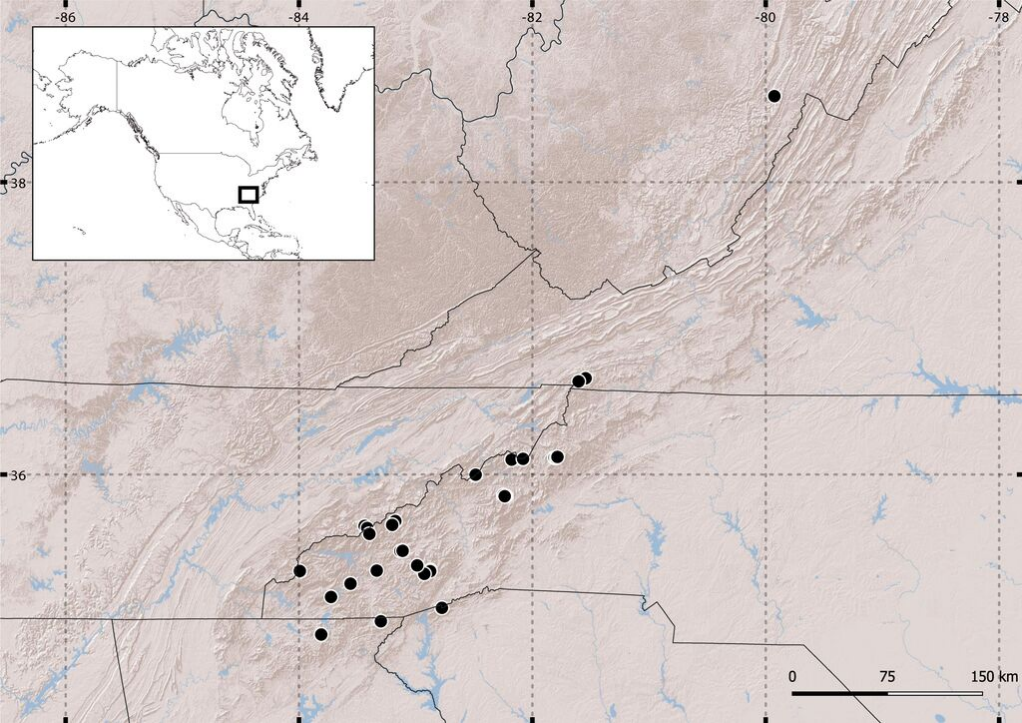
Sampled localities in the southern Appalachian Mountains and West Virginia.

After the sample sorting, we selected a total of 70 specimens from 25 localities in the southern Appalachian Mountains plus one further north from an Appalachian locality in West Virginia (Fig. 1). Of these 71 specimens, we amplified the barcoding fragment for 67 specimens, with a success rate of 94.4%) (Suppl. material 1) and, amongst the latter, we recovered 54 vouchers for morphological study (81%) (Suppl. material 2).

**Figure 2.**
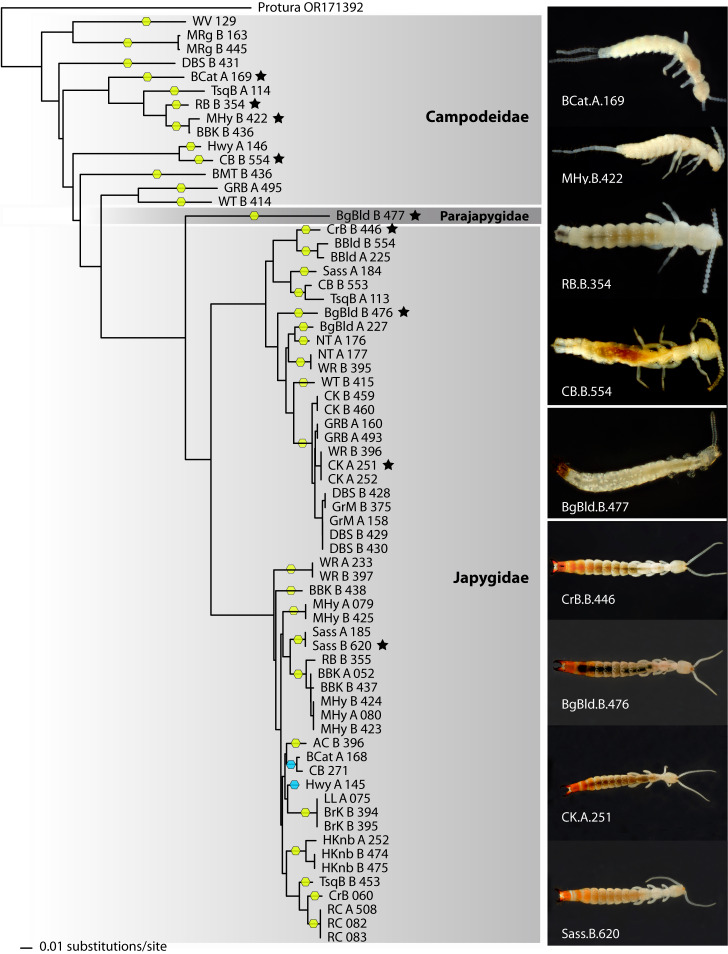
Neighbour-joining tree of Appalachian Diplura, based on K2P distances. Hexagons indicate ASAP delimited species (blue hexagons indicate a single delimited species not clustered together in NJ analysis). Asterisks denote specimens whose vouchers are shown at the right.

Sequences corresponding to specimens of the family Japygidae form a well-defined cluster in the NJ tree, divided into two main groups (Fig. 2), observed also in the ML analysis (Suppl. material 3). The single Parajapygidae barcode is placed as sister to the Japygidae clade in the NJ tree (Fig. 2) and, in the broader ML analysis, it resolves together with other Parajapygidae, even if, with the analysed data, the family does not form a single, resolved clade. The sequences corresponding to the Campodeidae do not form a single, monophyletic clade in any of our analyses, but are grouped in several smaller clades. The data available in GenBank and BOLD are not enough to assign any of our specimens to species or even genus and, in fact, several genera appear as polyphyletic in our ML analyses.

**Figure 3.**
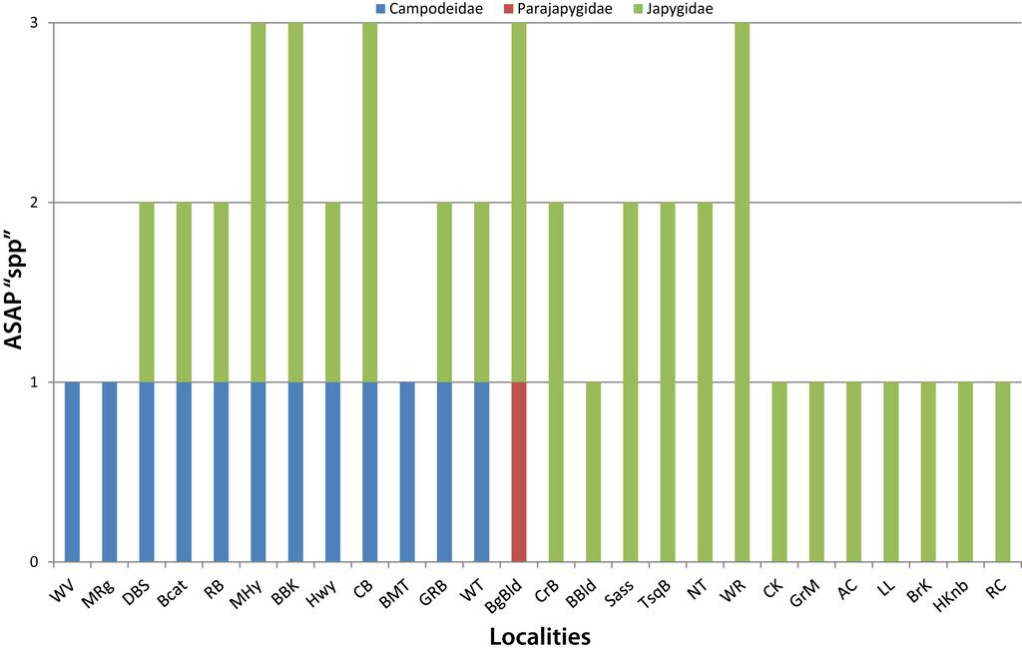
Number of ASAP-estimated Diplura species per family in each sampled locality.

The ASAP analysis resulted in a best partition suggesting the existence of 35 potential species, selecting a distance threshold of 2.3%. According to these results, the Campodeidae would be represented by 12 species, 22 of Japygidae and one species of Parajapygidae. Most sampled localities harbour a single species (11 localities) or two (10 localities), with a maximum of three species (five localities) (Fig. 3). We found no more than one campodeid species per locality, while Japygidae were represented by up to three species. Most ASAP-delimited campodeid species are exclusive to a single locality; only one (Dipura-003) was found in two different localities of the same range, the Great Balsams in North Carolina. Amongst the Japygidae, a majority of species was also found at a single locality, with one present at two localities of the Great Balsams (Diplura-002), another at two localities of the Plott Balsams (Diplura-012) and another on two balds in the Nantahala National Forest (Diplura-013). However, we found a few cases of species present in different ranges: one with presence in two localities in the Great Smoky Mountains as well as at one bald in the Nantahala National Forest (Diplura-006), one with populations in the Black Mountains and on Grandfather Mountain (Diplura-026) and another present in the Black Mountains (two localities), the Roan Highlands and on Grandfather Mountain (Diplura-015). In any of these cases, the maximum straight-line distance between populations of a single species is around 50 km (Diplura-015). No species is distributed on both sides of the main biogeographic barrier in the region, the Asheville Depression and only Diplura-013 crosses another important barrier, the Little Tennessee River Basin.

Mean uncorrected p-distances are shown in Suppl. material 4. Only two pairs show distances below 3% (Diplura-016/017 and Diplura-008/025) and six pairs below 4% (Diplura-001/023, Diplura-004/006, Diplura-006/029, Diplura-008/026, Diplura-016/031 and Diplura-017/031). Mean intraspecific distances for those delimited species with more than one barcode ranged from 0 to 2.14%.

## Discussion

According to our results, the diversity of Diplura in the southern Appalachian Mountains could be higher than currently known. The number of delimited species is 2 times higher than the number of known species in the Campodeidae and 3.7 times higher in the Japygidae. Additionally, although rare (one specimen), we report the presence of members of the Parajapygidae for the first time in these mountains, although this could be expected since two genera are known from neighbouring areas: *Miojapyxamericanus* Ewing 1941 from South Carolina, *Parajapyxunidentatus* (Ewing) 1941 from Alabama and *Parajapyxscalpellus* Fox 1941 from Georgia and North Carolina (Allen 2002). These diversity results must be interpreted with some caution as ASAP species delimitation results may deviate from the real number of species either overestimating (e.g. Hupało et al. 2022, Recuero and Caterino (2024b)) or underestimating it (e.g. Ranasinghe et al. (2023), Schattanek-Wiesmair et al. (2024)), although studies on a diverse array of organisms has shown ASAP to be one of the most reliable single-locus species delimitation methods (e.g. Muster et al. (2021), Serrano and Ortiz (2023), Solovyeva et al. (2023), Yin et al. (2023), Schattanek-Wiesmair et al. (2024)). With very few molecular studies on Diplura including different populations of the same species, there is little information on the typical intraspecific genetic distances in Diplura. A cave Campodeidae species, *Cestocampaiberica* Sendra & Condé, 2012, presenting isolated populations in central and eastern Iberia, shows intraspecific distances ranging from 1 to 9% (Sendra et al. 2012), while intraspecific divergence in some Chinese parajapygid species ranged from 1.5 to 5.3% with a mean of 1.9% (Bu et al. 2012). Considering these values, our delimitations could be overestimated as they are based on a distance threshold of 2.3%, but it is also possible that those studies may be including multiple cryptic species not yet delimited. However, in our ASAP analysis, models considering larger distance thresholds are considerably less supported. In addition, most of the genetic distances between delimited species pairs are much higher than that threshold, with only two pairs showing distances below 3% and six pairs below 4%, so we consider that the proposed species hypothesis should be close to the real diversity.

It was impossible to identify any of the southern Appalachian barcodes to species or even genus using the available sequences in GenBank and BOLD. This is not surprising, since the barcoding databases for soil arthropods in this region are largely incomplete (Recuero et al. 2023). Hopefully, the material presented here will soon be studied by Diplura taxonomists, improving the resolution of such databases as already done for other groups (e.g. Caterino (2022), Recuero and Caterino (2023)). It was more surprising to find that some genera, like *Campodea* and *Plusiocampa*, formed no monophyletic clades in our ML analysis. This could be, indeed, an artefact from the analysed data, a small fragment from a single mtDNA locus, which may be not enough to resolve those relationships correctly; but it could also indicate other problems like misidentifications or contaminations of deposited sequences or even the need for a systematic revision of those groups.

All delimited species present limited geographic distributions and most of them are restricted to a single locality or to nearby mountains within the same range. Even for the most widespread species, the japygid Diplura-015, populations are not separated by more than 50 km. In this sense, most species have not been able to cross some of the main biogeographic barriers, with only one case of presence across the Little Tennessee River Basin barrier. Sympatry of multiple dipluran species is not rare (Lock et al. 2010, Graening et al. 2014), but, contrary to the mentioned studies, we have not recovered sympatry amongst campodeid species in the southern Appalachians. We are not sure whether this is a real pattern in this region or it is a methodological artefact, as it is possible that, given the small size and morphological uniformity amongst the collected species, we failed to recover every campodeid morphospecies from each sampled locality and it is possible that even more different species are to be found in these forests. These insects are indeed rare, as the samples reported here contained only 176 total specimens or an average of only 2.4 specimens per morphospecies (most were singletons and only a few japygids were represented by ~ 10 specimens in a sample).

Although constrained by sampling limits, a few broader scale biogeographic patterns are evident. Both of the two main lineages of Japygidae traverse the Asheville Depression. In one lineage, only a single species, represented by two sequences from Woody Ridge Trail in the Black Mts (WR.A.233 and WR.B.397), occurs northeast of this biogeographic feature and is sister to an otherwise entirely south-western group. In the other main lineage, a smaller south-western lineage (BBld, Sass, TsqB) is sister to a predominantly north-eastern one, distributed in all the major ranges from Big Bald and the Roan Highlands, through the Black Mountains, Grandfather Mt. and on up into the Grayson Highlands of southern Virginia. Sampling in the Campodeidae was much sparser and no such clear patterns are evident there.

In general, the diversity of Diplura in the southern Appalachian Mountains seems to be higher than currently known, though not as high as in other small Hexapoda groups living in the litter of the same forests according to similar data and species delimitation methods (Dukes et al. 2022, Caterino and Recuero 2023). Even if the USA is amongst the better-studied regions for Diplura, still few taxonomic studies have been published in the last few decades (Thibaud et al. 2022). The data presented here should be integrated into detailed taxonomic accounts that will give us a more precise idea on the real diversity of this relevant hexapod group in the soils and litters of the southern Appalachian forests.

## Supplementary Material

ACC51C94-F060-5508-AABE-DCE4F1C7B74D 10.3897/BDJ.12.e125162.suppl1 Supplementary material 1 Cytochrome Oxidase I barcode region sequences for Appalachian Diplura Data type phylogenetic Brief description A nexus file including 67 aligned partial COI sequences of Diplura from the southern Appalachians. File: oo_1022644.nex https://binary.pensoft.net/file/1022644 Caterino MS, Recuero E

AFFA6E8D-D9B6-591C-9815-58936054035C 10.3897/BDJ.12.e125162.suppl2 Supplementary material 2 Voucher and collecting information for Diplura barcode sequences Data type occurrence Brief description An Excel spreadsheet containing specimen collecting data (locality, date, lat/long), voucher codes, DNA extraction codes and GenBank accession numbers for all Diplura sequences reported. File: oo_1022641.xlsx https://binary.pensoft.net/file/1022641 Caterino MS, Recuero E

0D3F8269-EAA8-5B88-B6F7-CC55AD47394F 10.3897/BDJ.12.e125162.suppl3 Supplementary material 3 Maximum Likelihood tree including southern Appalachian, GenBank and BOLD barcodes of Diplura Data type phylogenetic Brief description Maximum Likelihood tree obtained from southern Appalachian, BOLD and GenBank COI barcodes of Diplura, generated using IQtree. Node support is measured with ultrafast bootstrap. File: oo_1022645.pdf https://binary.pensoft.net/file/1022645 Caterino MS, Recuero E

502BE15D-BD38-5641-9C1E-5086CB6BC51F 10.3897/BDJ.12.e125162.suppl4 Supplementary material 4 Mean COI uncorrected p-distances between ASAP-delimited species of Diplura and intraspecific distances for species with more than one sequence (in percentage). Data type phylogenetic Brief description An Excel spreadsheet with mean COI uncorrected p-distances between ASAP-delimited species of Diplura and intraspecific uncorrected distances. File: oo_1022647.xlsx https://binary.pensoft.net/file/1022647 Caterino MS, Recuero E
