# Supplementary figures and images for: Molecular diversity of Diplura in southern High Appalachian leaf litter
Source: Biodivers Data J. 2024 May 27;12:e125162. doi: 10.3897/BDJ.12.e125162 (PMC11150871; doi:10.3897/BDJ.12.e125162)

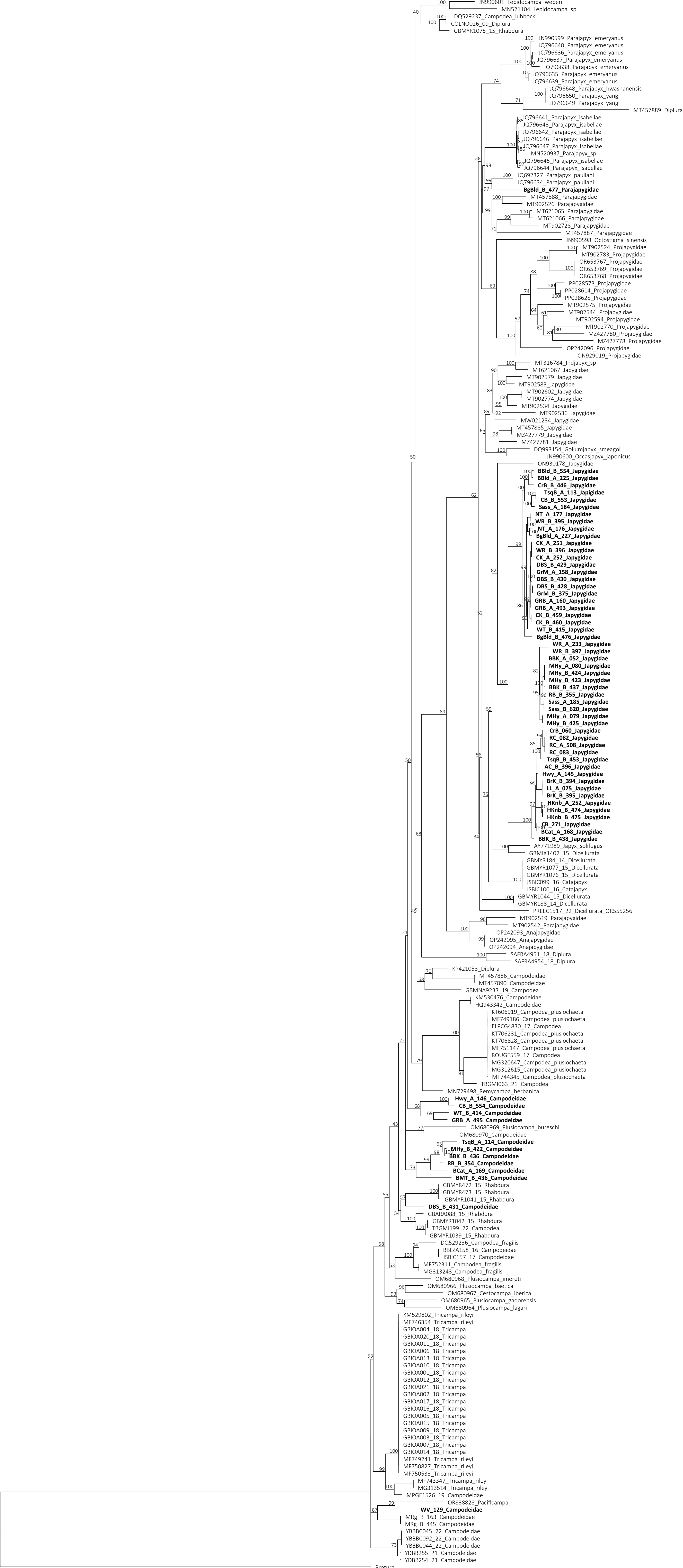

Supplement: Supplementary material 3 — Maximum Likelihood tree including southern Appalachian, GenBank and BOLD barcodes of Diplura [file bdj-12-e125162-s003.pdf]
